# How Does B-Value Affect HARDI Reconstruction Using Clinical Diffusion MRI Data?

**DOI:** 10.1371/journal.pone.0120773

**Published:** 2015-03-24

**Authors:** Sangma Xie, Nianming Zuo, Liqing Shang, Ming Song, Lingzhong Fan, Tianzi Jiang

**Affiliations:** 1 Brainnetome Center, Institute of Automation, Chinese Academy of Sciences, Beijing, China; 2 National Laboratory of Pattern Recognition, Institute of Automation, Chinese Academy of Sciences, Beijing, China; 3 Key Laboratory for NeuroInformation of Ministry of Education, School of Life Science and Technology, University of Electronic Science and Technology of China, Chengdu, China; 4 The Queensland Brain Institute, the University of Queensland, Brisbane, QLD, Australia; 5 CAS Center for Excellence in Brain Science, Institute of Automation, Chinese Academy of Sciences, Beijing, China; University of Minnesota, UNITED STATES

## Abstract

**Background:**

A number of imaging factors can affect the orientation distribution function (ODF) reconstruction in high angular resolution diffusion imaging (HARDI). The aim of this study was to investigate the effect of the b-value on the HARDI reconstruction and to seek for the appropriate b-value for ODF reconstruction from clinical HARDI data.

**Methods:**

Diffusion MRI data with various b-values were collected on a GE 3T MRI scanner. To reconstruct the diffusion ODF and fiber ODF, decomposition-based spherical polar Fourier imaging and deconvolution-based constrained spherical deconvolution approaches were applied separately. The full width at half maximum (FWHM) of the ODF and the angular difference of the peaks extracted from ODF were measured to investigate the effect of b-value on the ODF reconstruction. Visual inspection of the ODF was used to evaluate the reconstructions.

**Results:**

The FWHM of the ODFs in the corpus callosum, which was chosen as the region of interest (ROI), decreased with increasing b-values. The differences in the FWHM for the diffusion ODF and the fiber ODF between the b-values of 2000 s/mm^2^ and 2500 s/mm^2^ were not significant. The angular differences of the ODF between 2000 s/mm^2^ and 2500 s/mm^2^ were lowest in both single-directional and two-directional situations. The ODFs became sharper and crossing-fiber situations were detected with an increase in b-value. B = 2000 s/mm^2^ and above revealed most of the two-way or three-way crossing-fiber structures.

**Conclusions:**

Considering both the signal-to-noise ratio and the acquisition time, b = 2000 s/mm^2^ is the basic requirement for ODF reconstruction using current HARDI methods on clinical data. This study can provide a useful reference for researchers and clinicians attempting to set appropriate scan protocols for specific HARDI experiments.

## Introduction

In recent years, imaging neuroscience has examined the human brain as a hierarchical and self-organizational network system [1, 2]. A variety of MRI modalities, including structural MRI [3], diffusion MRI [4, 5], and functional MRI [6], have been exploited to characterize the structures or activities of the brain on different tempo-spatial imaging scales [7]. Although the performance of these techniques leaves much to be desired, each of them has provided a specific perspective for investigating how the brain works [7].

Diffusion MRI (dMRI) is a non-invasive imaging technique that, to date, is unique in that it can be utilized to reveal the microstructure of the white matter of the in-vivo human brain [8]. In the three decades since the emergence of dMRI, this technique has evolved through two major steps. One is traditional diffusion tensor imaging (DTI), for which a Gaussian model was adopted to represent the distribution of water molecules [9]. Although the first step, DTI, is not able to handle crossing-fiber microstructures, such as fanning, kissing and multi-crossing, the second step, HARDI, is able to partially solve such multiple intravoxel fiber populations. Except for some extensions of diffusion tensor models, such as the multi-Gaussian model [10] and the ball-stick model [11], most of the researchers using the HARDI method have used one of three general approaches: diffusion spectrum imaging (DSI) [12], decomposition-based methods [11, 13–18], and deconvolution-based methods [19–21]. Because the DSI method usually requires dense sampling in q-space, it is seldom utilized in clinical data analysis. Instead, the other two methods have received increasing attention in the recent literature. Among the decomposition-based methods, the spherical harmonic function is a good choice [11, 13, 16–18]. Assemlal et al. [14] proposed orthonormal basis Spherical Polar Fourier (SPF) as the basis for a new method for revealing tissue micro-architecture. Subsequently, Cheng et al. [15] proposed an analytical solution for the above method for clinical applications. Deconvolution-based methods have been proposed by many researchers [19–21] as ways to estimate the fiber ODF directly rather than indirectly through the diffusion ODF. For example, Tournier et al. [20] introduced a constraint on the negative regions in the fiber ODF to improve the spherical deconvolution. The information content of the diffusion ODF is strongly related to that of the fiber ODF. Nonetheless, they provide different types of information. The diffusion ODF represents the motion of water molecules, which often, but not exclusively, diffuse parallel to the fiber, whereas the fiber ODF describes the distribution of the nerve fiber directions within a voxel [22]. This implies that the fiber ODF will be sharper than the diffusion ODF.

However, a number of imaging parameters can affect both the local reconstruction accuracy and the subsequent global connectivity analyses [23, 24]. These parameters include repetition time (TR), echo time (TE), b-value, the number and distribution of the gradient directions, and the imaging voxel size [25]. Extremely high b-values and long acquisition times are not acceptable in clinical situations. A set of appropriate acquisition parameters that can lead to acquiring a local reconstruction that has adequate quality is of major importance in clinical research. The b-value is one of the main factors that can affect the ODF reconstruction, yet most clinical MRI scanners only allow it to be adjusted over a certain range. Thus it has received an enormous amount of attention and has been investigated from a number of perspectives [23, 26–29]. Alexander and Barker [23] used Monte Carlo simulations to investigate the optimal b-value for estimating white matter fiber orientation with a standard spherical sampling scheme and found that the optimal b-values were in the ranges of 700–1000 s/mm^2^ and 2200–2800 s/mm^2^ for one-fiber and two-fiber situations, respectively, in brain tissue. Meanwhile, phantom data and human brain data were also used to evaluate the accuracy of the q-ball imaging (QBI) method [30]. These revealed that the accuracy of QBI was worse at higher b-values and that the angular resolution was highly dependent on the b-value [26]. Tournier et al [29] focused on single-fiber voxels and the angular frequency content of the diffusion weighted signal itself to evaluate the minimum number of diffusion weighted directions and optimal b-values required for HARDI. However, when using the two representative types of HARDI methods, that is, decomposition-based methods such as spherical polar Fourier imaging (SPFI) [14, 15] and deconvolution-based methods such as constrained spherical deconvolution (CSD) [20], establishing the appropriate b-value choice for the ODF reconstruction using clinical HARDI data is essential. In particular, because of HARDI’s usefulness in newly emerging human brain projects [31, 32], a reference that establishes a reasonable b-value choice could play a critical role in clinical HARDI data acquisition and post processing.

In this study, we used in-vivo human brain data collected by clinical imaging protocols with multiple b-values to reconstruct the diffusion ODFs and fiber ODFs in single-fiber and crossing-fiber regions, using decomposition-based analytical SPFI and deconvolution-based CSD approaches separately. Then we quantitatively and qualitatively evaluated the results of each reconstruction. Please note that our intention was not to compare the performance between the SPFI and the CSD, since they belong to different categories of HARDI methods, but instead was to examine the effect of the b-value on the ODF when it was reconstructed utilizing these two representative HARDI methods (the SPFI and the CSD) using clinical data. We believed that such comprehensive comparisons would provide a reasonable and useful reference for researchers and clinicians to allow them to set adequate b-values for specific clinical HARDI data acquisitions and analyses.

## Materials and Methods

### 2.1 MRI data acquisition and preprocessing

Human dMRI acquisition was performed on eight healthy subjects using a twice refocused spin-echo echo-planar imaging sequence on a GE Discovery 750 3T MRI scanner at the University of Electronic Science and Technology of China. The study was approved by the ethics committee of the University of Electronic Science and Technology of China. The participants’ written informed consent was obtained prior to the scanning. The main parameters included: field-of-view (FOV) = 256 * 256 mm^2^, matrix size = 128 * 128, slice = 75, voxel size = 2 * 2 * 2 mm^3^ and without a slice gap. The datasets were acquired with 64 noncollinear diffusion gradient directions separately at b-values of 650, 1000, 1500, 2000, 2500 s/mm^2^. For each b-value, three images with b = 0 s/mm^2^ were also acquired. The b-value is characterized as $b=\gamma^{2}G^{2}\delta^{2}\left(\Delta -\frac{\delta }{3}\right)$, where γ is the gyromagnetic ratio, *G* is the amplitude of the magnetic field gradient pulses, δ is the gradient pulse duration and *Δ* is the temporal separation. In this study, TE was set by the clinical scanner according to the selected b-value. The maximum gradient amplitude used in this acquisition was 40 mT/m. For data preprocessing, we performed eddy current corrections on all the diffusion-weighted images using eddy_correct in FSL 5.0 [33] by first aligning the b0 volume of each session to the first b0 volume then registering each diffusion-weighted volumes to the b0 image of the current session. The corpus callosum and the centrum semiovale, which were used as ROIs in this study, were not affected by echo-planar imaging induced distortion.

### 2.2 Measurement of SNR

The signal-to-noise ratio (SNR) was measured using a two-region method [34] and is defined as in Eq. 1&2. We computed the average SNR across all gradient direction diffusion weighted imaging (DWI) volumes of each b-value separately to represent the SNR of the subject and calculated the SNR for all eight subjects. $S_{b-value}^{g_{i}}\left(ROI\right)$ stands for the signals of the voxels in the ROI of the *i*th gradient direction DWI volume, and $S_{b-value}^{g_{i}}\left(background\right)$ stands for the signals of the voxels in the background ROI outside the brain of the *i*th gradient direction DWI volume. We selected the corpus callosum and centrum semiovale as the ROIs for calculating the mean signals and a region outside the brain as the background ROI to calculate the standard deviation of the noise. Finally, the SNR of the diffusion-weighted data at each b-value was calculated to investigate the trend in the SNR with increasing b-value.

$$SNR_{b-value}=mean({\text{SNR}}_{b-value}^{g_{1\cdots N}})$$ (1)

$$SNR_{b-value}^{g_{i}}=\frac{mean\left[S_{b-value}^{g_{i}}\left(ROI\right)\right]}{sd\left[S_{b-value}^{g_{i}}\left(background\right)\right]}$$ (2)

### 2.3 SPFI reconstruction

In SPFI, which was first proposed by Assemlal et al. [14], the diffusion signal E(*q*) is represented by the spherical polar Fourier (SPF) basis functions in Eq. 3.

$$E\left(q\right)\;=\;\prod_{n=0}^{N}\sum_{l=0}^{L}\sum_{m=-l}^{l}a_{lmn}R_{n}(\left\|q\right\|)Y_{l}^{m}(u)$$ (3)

The SPF basis denoted by $R_{n}(\left\|q\right\|)Y_{l}^{m}(u)$ is a 3D orthonormal basis with spherical harmonics in the spherical portion and the Gaussian-Laguerre function in the radial portion. Furthermore, Cheng and his colleagues proposed an analytical solution for transforming the coefficients *a*_*nlm*_ of E(*q*) to the coefficients $c_{lm}^{\Phi_{w}}$ of the diffusion ODF represented by the spherical harmonics basis, as in Eq. 4 [15].

$$\Phi_{w}\left(r\right)=\sum_{l=0}^{L}\sum_{m=-l}^{l}c_{lm}^{\Phi_{w}}Y_{l}^{m}\left(r\right)$$ (4)

This is a model-free, regularized, fast and robust reconstruction method which can be performed with single-shell or multiple-shell HARDI data to estimate the diffusion ODF proposed by Wedeen et al. [12]. The reconstruction of the ODF using analytical SPFI includes two independent steps. The first estimates the coefficients of E(*q*) using a least squares method, and the second transforms the coefficients of E(*q*) to the coefficients of the diffusion ODF. The reconstruction method was implemented using an in-house software written in C++. In this study, the maximum angular order was 4, and the maximum radial order was 1. The angular and racial regularization parameters were both 10^−8^.

### 2.4 CSD reconstruction

The CSD method proposed by Tournier et al. [20, 21] expresses the diffusion signal as in Eq. 5,

$$S\left(\theta ,\Phi \right)=F\left(\theta ,\Phi \right)⊗R\left(\theta \right)$$ (5)

where *F*(*θ*, Φ) is called the fiber orientation density function (fiber ODF), which needs to be estimated, and *R*(*θ*) is the response function, which is the typical signal generated by one fiber. The response function can be directly estimated from DWI data by measuring the diffusion profile in the voxels with the highest fractional anisotropy (FA) values, indicating that a single coherently oriented fiber population is contained in these voxels. When the response function is obtained, we can utilize the deconvolution of *R*(*θ*) from S(*θ*, Φ) to estimate the fiber ODF. The computation of the fiber ODF was carried out using the software MRtrix (J-D Tournier, Brain Research Institute, Melbourne, Australia, http://www.brain.org.au/software/), and a maximum harmonic order *l*_max_ = 8 was used in this study [35].

### 2.5 FWHM measurement

The FWHM of the normalized ODF was measured by Cho and his colleagues to quantify the angular resolution in Q-ball imaging [26]. In this study, we calculated the FWHM of the diffusion ODF derived from the SPFI and the fiber ODF derived from the CSD to quantify the angular resolution. The calculation of the FWHM can be described as follows. First, we extracted the principal direction υ(*θ*_max_, *φ*_max_) of the ODF according to Eq. 6.

$$\upsilon \left(\theta_{\text{max}},\varphi_{\text{max}}\right)=\text{arg}\;{\text{max}}_{\text{υ}}\text{ODF}\left(\theta ,\varphi \right)$$ (6)

We used a mesh search on the spherical function to find potential local maxima by using a coarse mesh. Then we set the local maxima as the initialization points and utilized a gradient ascent method to update the maxima in continuous space.

Secondly, we assumed that the uniform fibers within each MR voxel were axially symmetric and consequently, that the ODF was an axially symmetric function [26]. Then we adjusted the zenith angle θ to increase the subtended angle between the current direction and the principal direction υ(*θ*_max_, *φ*_max_) from 0 to π. In this way we were able to obtain a plot of the ODF for angles from 0 to π. Finally, the FWHM was defined as in Eq. 7.

$$FWHM=2\left|\theta_{FWHM}-\theta_{\text{max}}\right|$$ (7)

**Fig 1:**
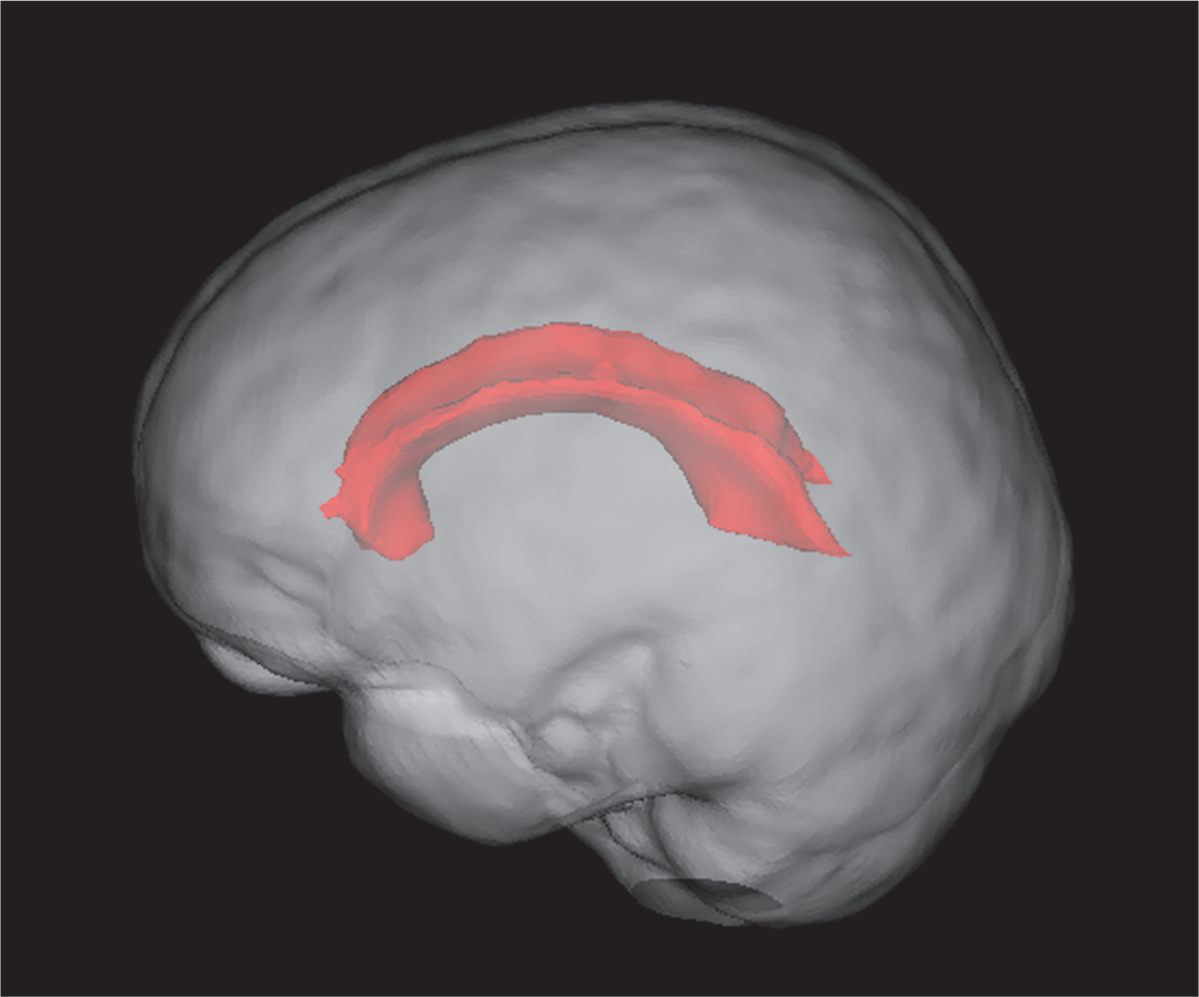
The corpus callosum used to calculate the FWHM.

Where *θ*_*FWHM*_ can be obtained using $ODF(\theta_{FWHM},\varphi_{\text{max}})=\frac{1}{2}ODF(\theta_{\text{max}},\varphi_{\text{max}})$. For the ROI used to measure the FWHM, we calculated the FA map with the DWI data of b = 1000 s/mm^2^ for each subject and selected the voxels with FA > 0.8 in the corpus callosum showed in Fig. 1 as the ROI to calculate the FWHM. We calculated the FWHM for each subject and averaged the FWHM across the voxels in the ROI for each individual to constitute the group results.

### 2.6 Measurement of angular difference

In order to quantify the stability of the peak(s) extracted from the ODF at increasing b-values, we calculated the angular difference of the principal direction(s) extracted from the ODF between different b-values. First, the number of principal directions and the peak orientations of each voxel in the white matter were calculated at each b-value for SPFI and CSD separately. The voxels that were identified by every b-value that contained only single direction fibers were selected as single-direction ROIs, and the voxels that were identified by every b-value that contained two-direction fibers were selected as the two-directional ROIs. Next, we separately measured the angular difference of the principal direction(s) of each voxel in the one-directional and two-directional ROIs. Since each voxel in the two-directional ROIs had two angular differences, we averaged them to calculate the angular difference for that voxel. We performed the calculation described above on each subject, and averaged the angular differences of all the voxels in the one-directional ROI and in the two-directional ROI separately as the angular differences for each subject.

### 2.7 Visual inspection of ODFs in complex fiber configurations

A visual inspection of the diffusion ODF and the fiber ODF was used as a qualitative assessment to investigate the effect of b-value on the reconstructed ODF and its ability to distinguish crossing-fibers using clinical data. We focused on the region that contains complicated fiber populations. The centrum semiovale, which contains three types of crossing fibers [corona radiate (CR), superior longitudinal fasciculus (SLF), and the striations of the corpus callosum (CC)] [26], has been widely used as the area of focus in studies assessing modeling results with respect to b-values. In this study, we qualitatively made a comparison of the ODFs in the centrum semiovale between different b-values.

### 2.8 Statistical analysis

The FWHM of Subject One (S1) at five b-values and the mean FWHM of each subject in the dataset were compared separately using analysis of variance (ANOVA) followed by Bonferroni’s post hoc tests when there were differences across the five groups. The significance threshold was set at *P* < 0.001, corrected. The data were analyzed using IBM SPSS Statistics 19.0 (Armonk, NY).

## Results

### 3.1 Relationship between SNR of DWI and b-value

**Fig 2:**
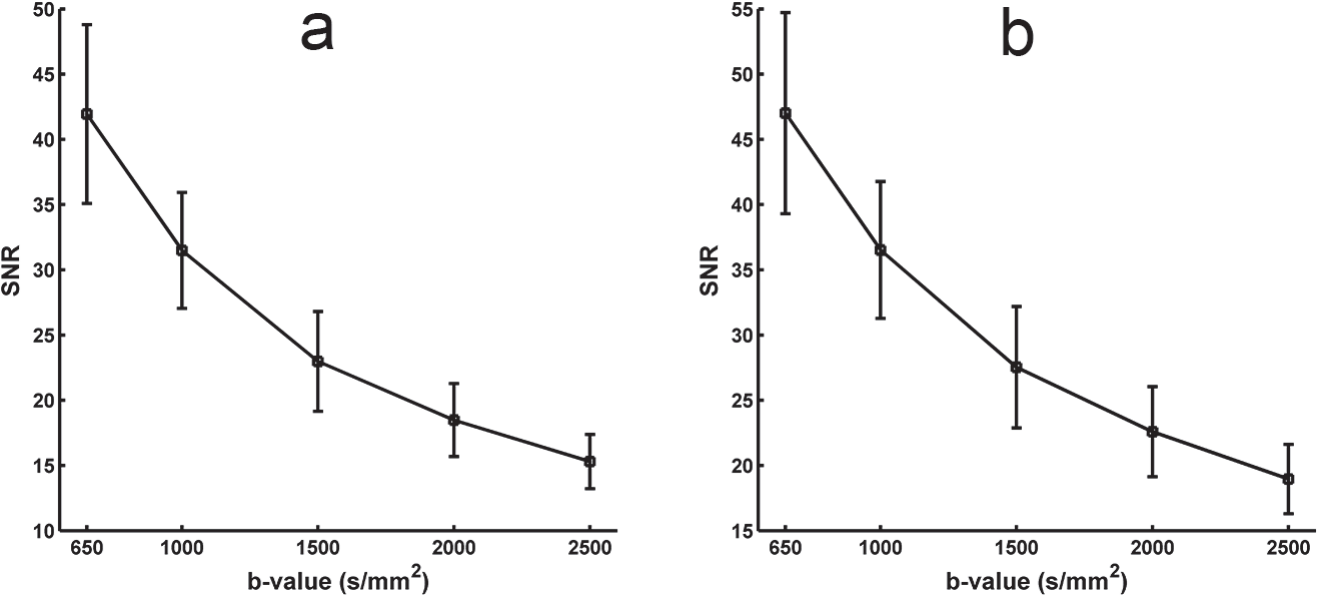
The relationship between the SNR and the b-value. (a) The SNR of corpus callosum. (b) The SNR of centrum semiovale.

The SNR of the DWIs at each b-value was calculated using a two-region method. Fig. 2 shows an inverse relationship between the SNR and the b-value. The highest SNRs of corpus callosum (Fig. 2A) and centrum semiovale (Fig. 2B) were 41.94 and 47.01 at b = 650 s/mm^2^ and the lowest SNRs were 15.30 and 18.96 at b = 2500 s/mm^2^.

### 3.2 FWHM of the diffusion ODF from SPFI

**Fig 3:**
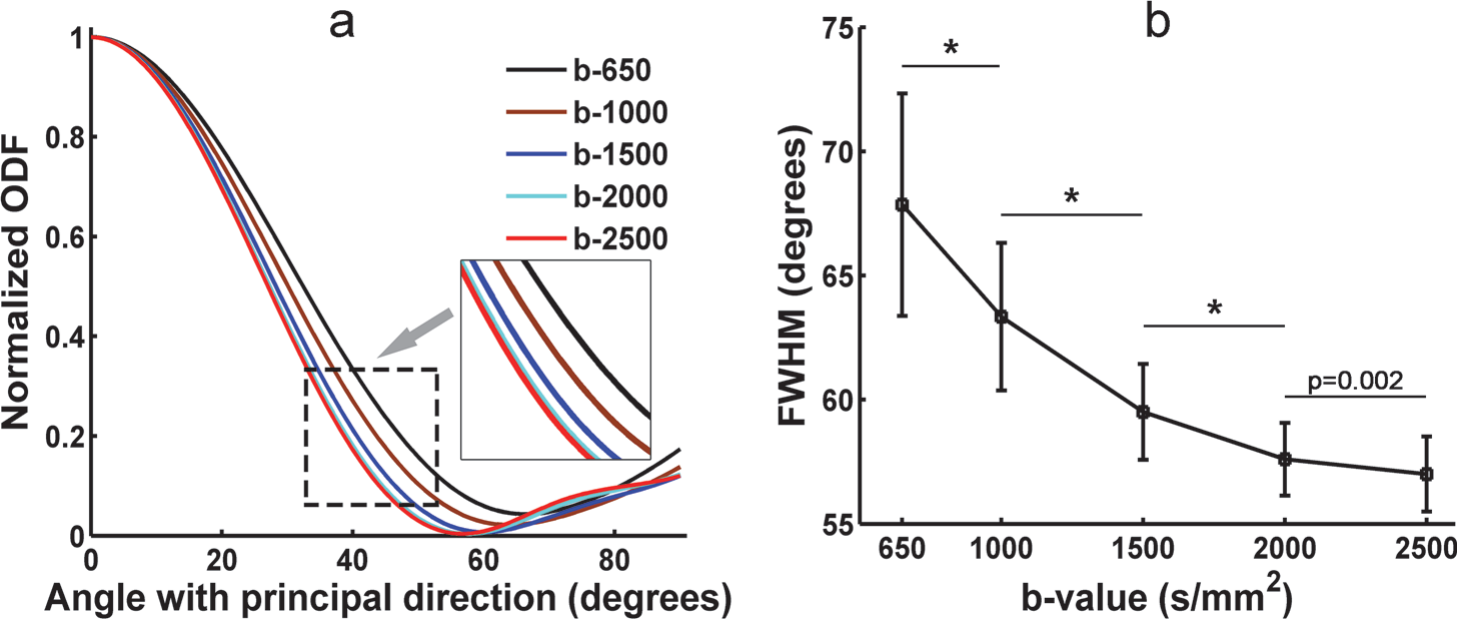
The FWHM of the diffusion ODF from S1 at five b-values. (a) Distribution of the diffusion ODF measured by SPFI for S1 at five b-values. The x-axis shows the colatitudes, where 0° indicates that the orientation is parallel to that of the principal direction. (b) The FWHM of the ODF profile measured in (a). (*) indicates statistically significant differences between groups (*p* < 0.001).

**Fig 4:**
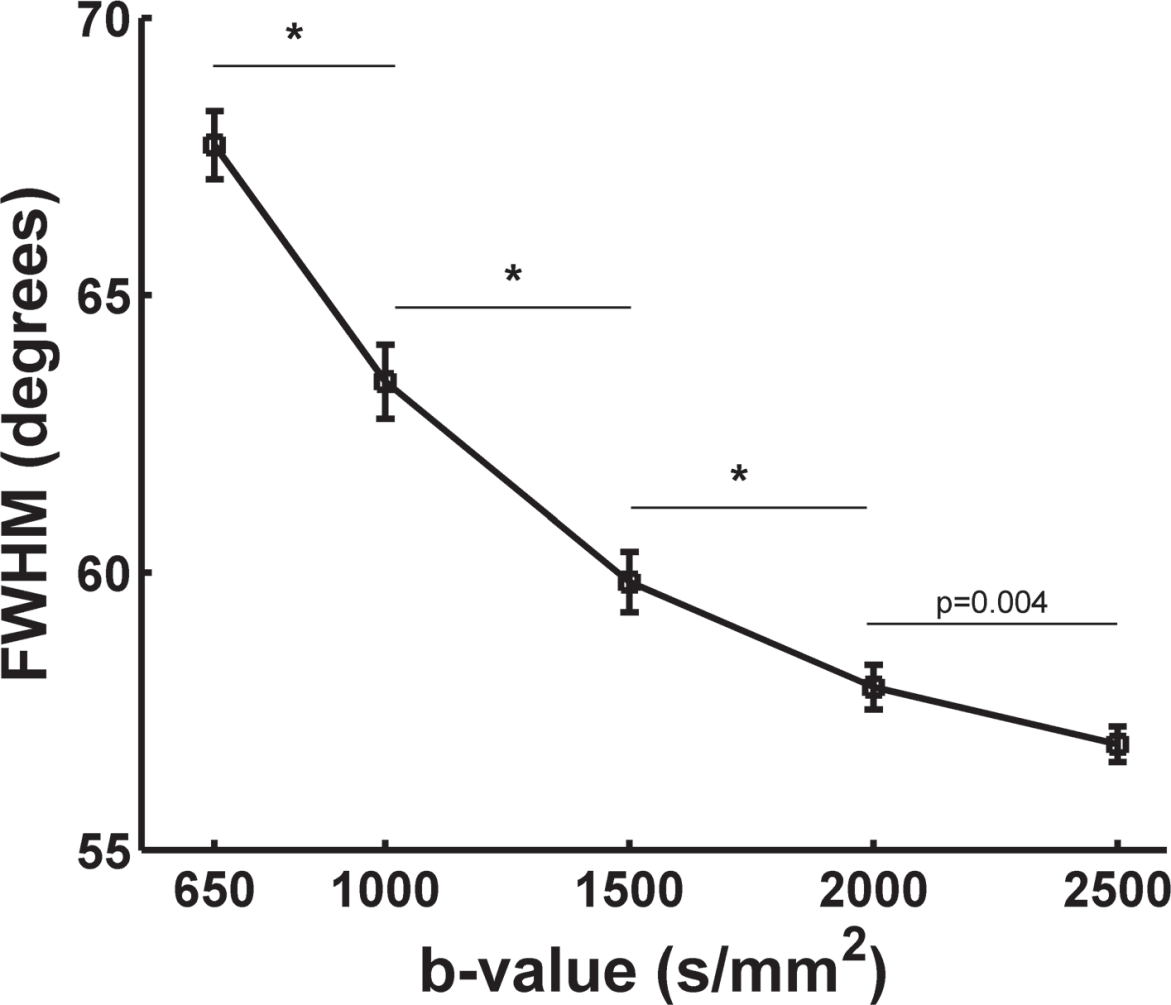
The group FWHM of the diffusion ODF from all eight subjects at five b-values. (*) indicates statistically significant differences between groups (*p* < 0.001).

In this study, the FWHM of the diffusion ODF was measured in the ROI that was located in the corpus callosum at five b-values for each subject. Fig. 3A illustrates the relationship between the contour of the main lobe and the subtended angle sliding along the contour of the main lobe at five b-values of Subject One (S1). Fig. 3B shows the FWHM of the diffusion ODF at five b-values of S1. Fig. 3A demonstrates that a higher b-value resulted in a lower FWHM and a sharper ODF. The profile of b = 2000 s/mm^2^ largely overlapped the profile of b = 2500 s/mm^2^. The FWHM was 67.9 at b = 650 s/mm^2^ and decreased to 57.0 at b = 2500 s/mm^2^. The difference in the FWHM between b = 2000 s/mm^2^ and b = 2500 s/mm^2^ was less than 1^o^. The FWHM of S1 decreased significantly from b-values of 650 s/mm^2^ to 1000 s/mm^2^, 1000 s/mm^2^ to 1500 s/mm^2^, and 1500 s/mm^2^ to 2000 s/mm^2^ (Fig. 3). There was no significant difference in the FWHM of S1 between the b-values of 2000 s/mm^2^ and 2500 s/mm^2^ (Fig. 3). Fig. 4 shows the group result for the FWHM of the diffusion ODFs. The group result was consistent with the result from S1. There were significant differences in the group FWHM between the b-values of 650 s/mm^2^ and 1000 s/mm^2^, 1000 s/mm^2^ and 1500 s/mm^2^, 1500 s/mm^2^ and 2000 s/mm^2^. No significant difference was found between the b-values of 2000 s/mm^2^ and 2500 s/mm^2^ (Fig. 4).

### 3.3 FWHM of the fiber ODF from the CSD

**Fig 5:**
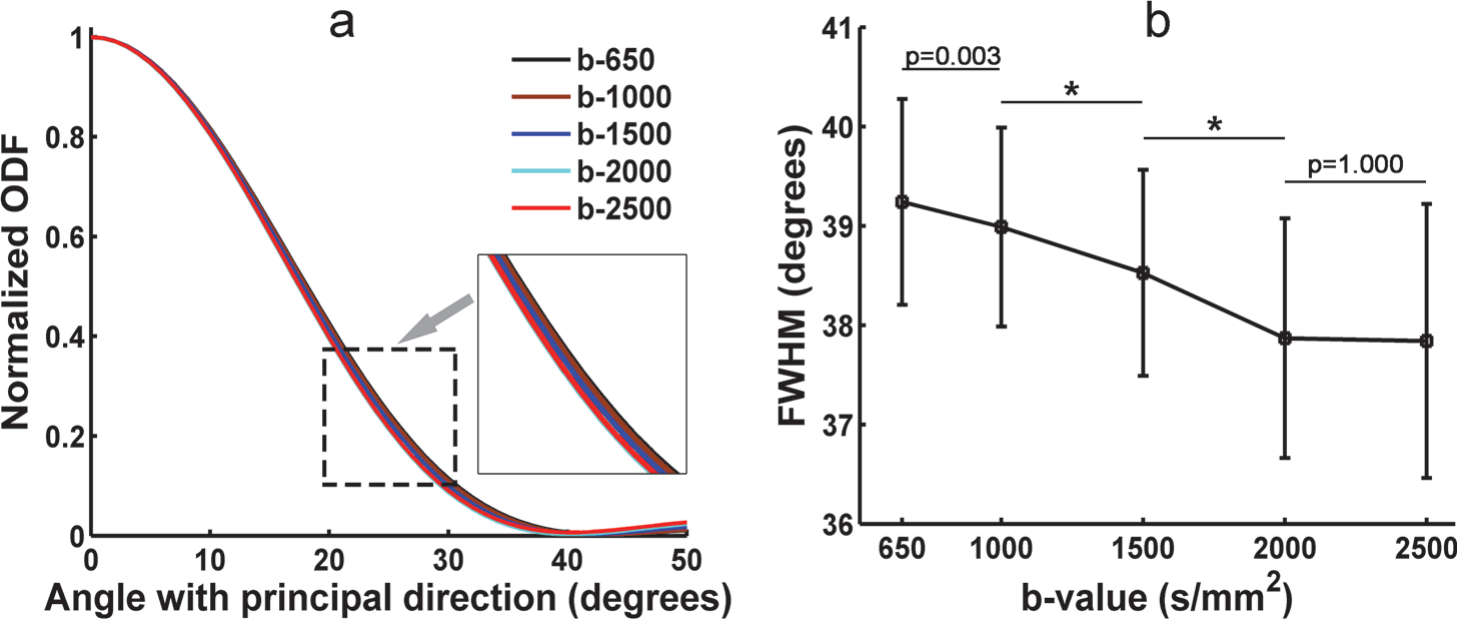
The FWHM of the fiber ODF from S1 at five b-values. (a) Distribution of the fiber ODF measured by CSD for S1 at five b-values. The x-axis shows the colatitudes, where 0° indicates that the orientation is parallel to that of the principal direction. (b) The FWHM of the ODF profile measured in (a). (*) indicates statistically significant differences between groups (*p* < 0.001).

**Fig 6:**
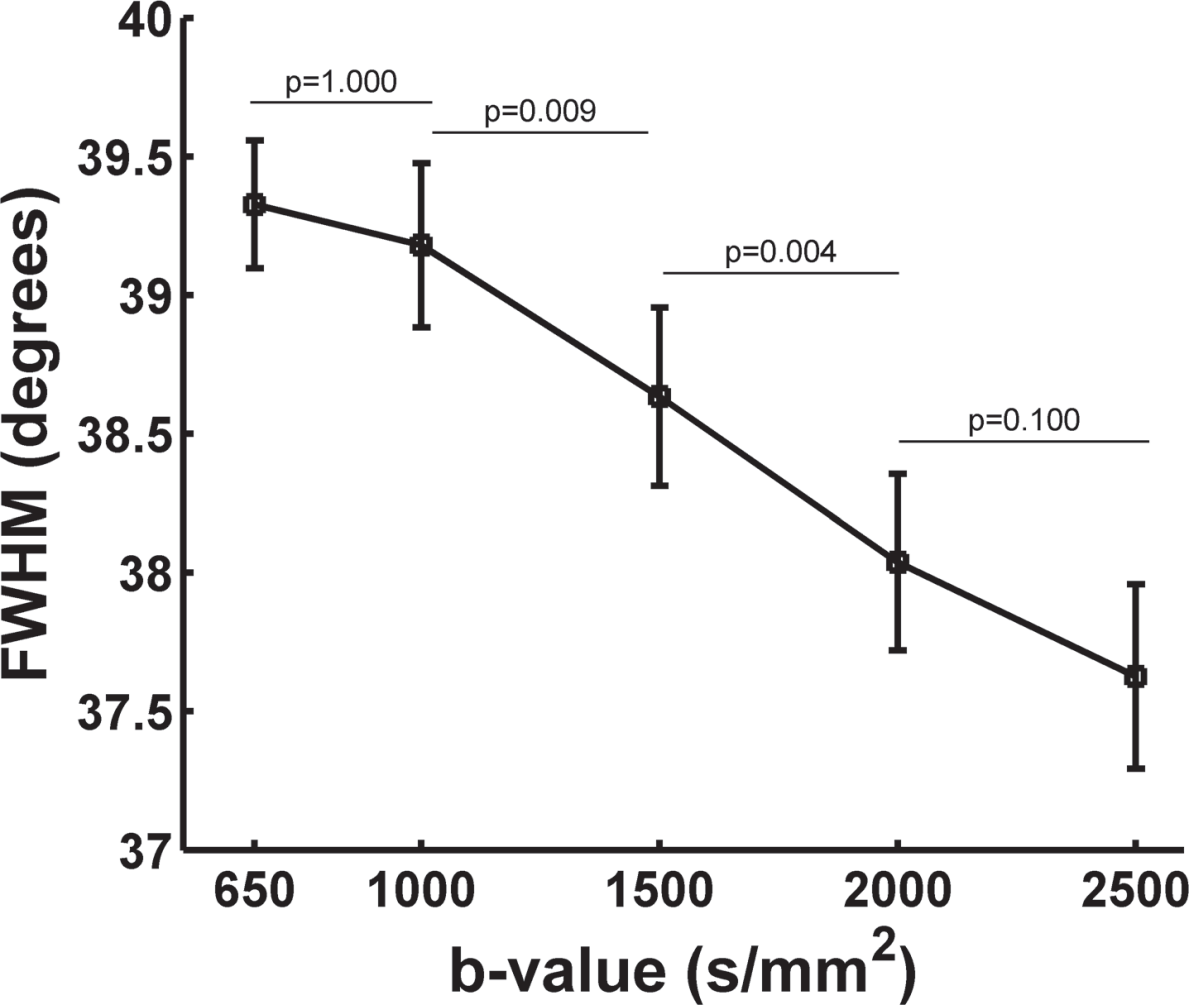
The group FWHM of the fiber ODF from all eight subjects at five b-values.

We also measured the FWHM of the fiber ODFs in the ROI which is located in the corpus callosum using the CSD at five b-values for each subject. Fig. 5A illustrates the relationship between the contour of the main lobe and the subtended angle sliding along the contour of the main lobe at five b-values of S1. We found no significant difference between the plots generated using different b-values of S1, but they aligned in the same sequence and followed the same trend as in Fig. 3A. Fig. 5B shows the FWHM of the fiber ODF at five b-values of S1. The tendency of the FWHM was consistent with the results from the SPFI (Fig. 3B). The FWHM was 39.2 at b = 650 s/mm^2^ and decreased to 37.8 at b = 2500 s/mm^2^, but the range of the change was narrower than the one found using the SPFI method. There were significant differences in the FWHM of S1 between the b-values of 1000 s/mm^2^ and 1500 s/mm^2^, 1500 s/mm^2^ and 2000 s/mm^2^ (Fig. 5). No significant difference in the FWHM of S1 was found between the b-values of 2000 s/mm^2^ and 2500 s/mm^2^ (Fig. 5). The group results of the FWHM of the fiber ODFs showed in Fig. 6 demonstrated a similar trend to that found for S1 and for the group results of the diffusion ODF. In the group FWHM, no significant differences were found between each b-value and its adjacent b-value, but, again, the data followed the same trend (Fig. 6).

### 3.4 Angular difference of the peaks extracted from ODFs based on different b-values data

**Fig 7:**
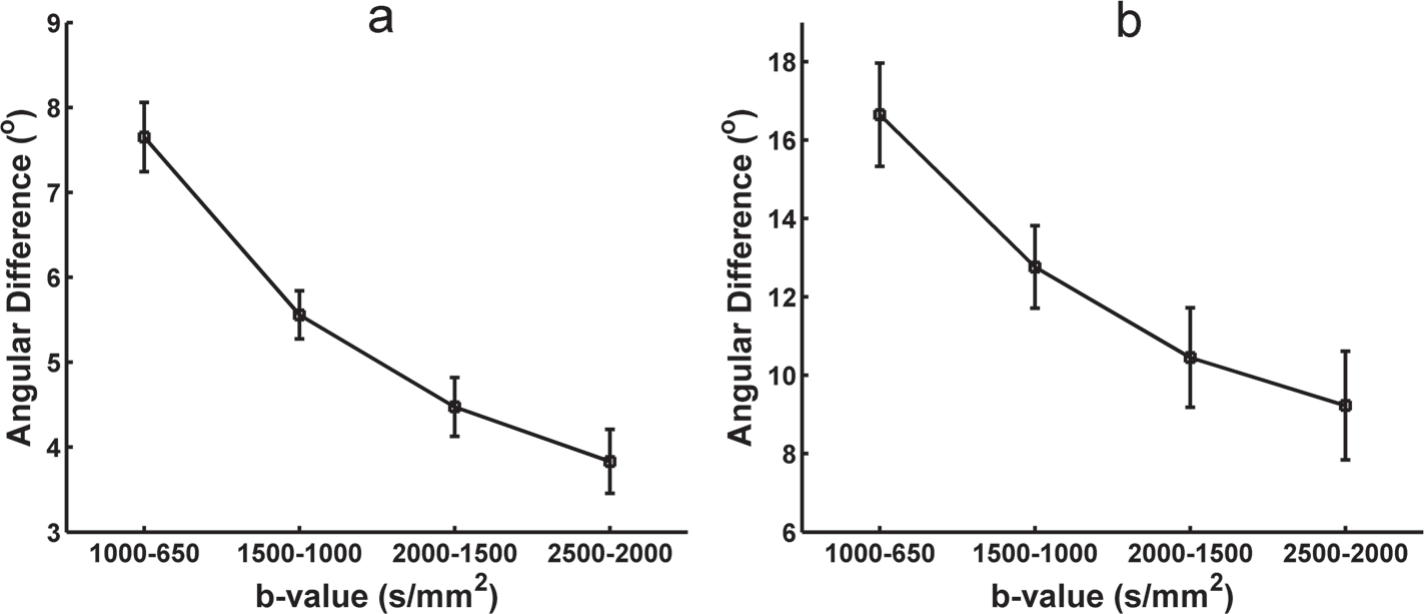
The angular difference in the diffusion ODF obtained with SPFI between each pair of neighboring b-values. (a) The angular difference for one-directional situations. (b) The angular difference for two-directional situations.

**Fig 8:**
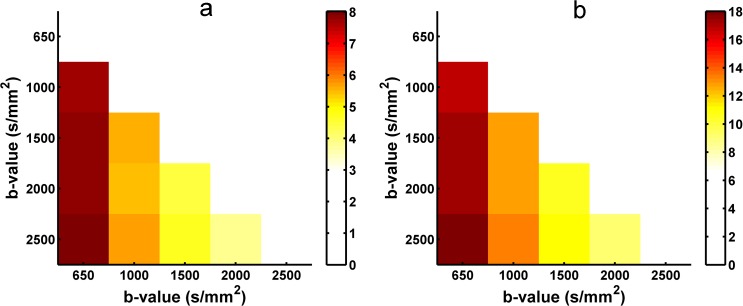
The angular difference in the diffusion ODF obtained with SPFI between each pair of b-values. (a) The angular difference for one-directional situations.(b) the angular difference for two-directional situations. The color in the grid indicates the angular difference in the ODF between the two b-values in the x-axis and the y-axis.

**Fig 9:**
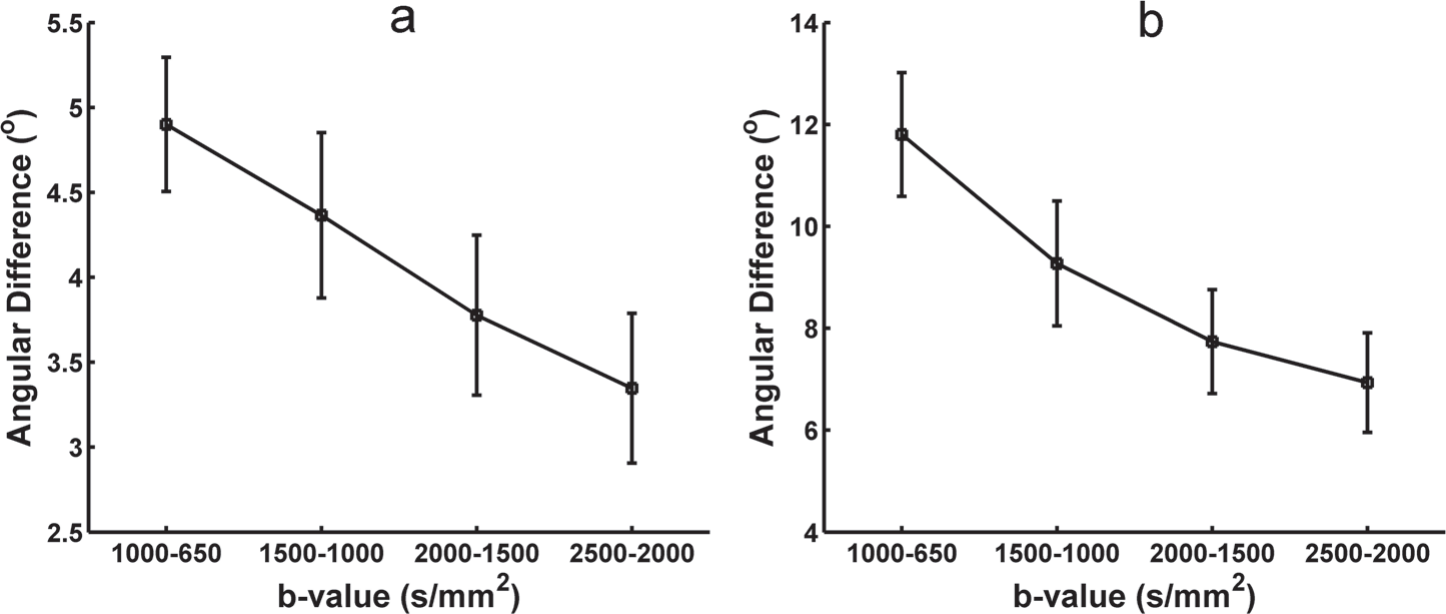
The angular difference in the fiber ODF obtained with CSD between each pair of neighboring b-values. (a) The angular difference for one-directional situations. (b) The angular difference for two-directional situations.

**Fig 10:**
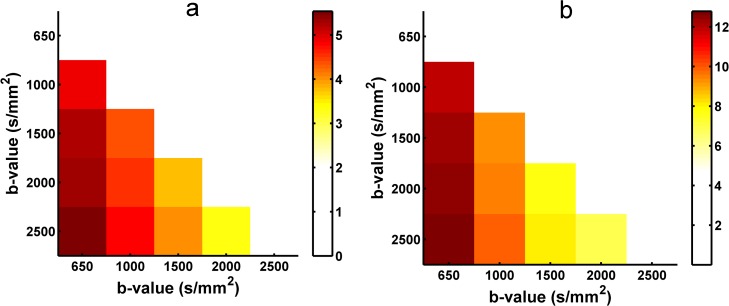
The angular difference in the fiber ODF obtained with CSD between each pair of b-values. (a) The angular difference for one-directional situations. (b) The angular difference for two-directional situations. The color in the grid indicates the angular difference in the ODF between the two b-values in the x-axis and the y-axis.

The angular difference in the principal orientations extracted from the diffusion and fiber ODFs using different b-values HARDI data was measured to quantify the stability of the reconstruction. Fig. 7 shows the angular difference of peaks extracted from the reconstructed diffusion ODFs between the two contiguous b-values. Both the one-directional (Fig. 7A) and two-directional situations (Fig. 7B) indicate that the angular difference becomes smaller and that the peaks of the diffusion ODF become more stable with higher b-values. Fig. 8 shows the angular difference in the primary orientations of the diffusion ODF between each pair of b-values for the one-directional (Fig. 8A) and the two–direction situations (Fig. 8B). The color of the grid represents the angular difference. The angular difference between b = 2000 s/mm^2^ and b = 2500 s/mm^2^ was the lowest out of all the angular differences for both the single-fiber and crossing-fiber situations (Fig 8A-B). In Fig. 8, the colors of the angular differences between the b-values of 1500, 2000 and 2500 s/mm^2^ are similar and show as a light yellow, which represents lower values. Figs. 9 and 10 show the angular difference results of for the fiber ODFs. The angular difference of the fiber ODFs between the two contiguous b-values decreased with the increasing b-value, and the overall trend was consistent with the result from the diffusion ODF. In the fiber ODF case, the difference between the b-values of 2000 s/mm^2^ and 2500 s/mm^2^ was also lowest for both single and crossing fiber situations. Fig. 10 illustrates that the angular differences for the fiber ODFs between the b-values of 1500, 2000 and 2500 s/mm^2^ were lower and more similar compared with the other pairs of b-values for both situations.

### 3.5 Diffusion ODFs in the centrum semiovale using SPFI

**Fig 11:**
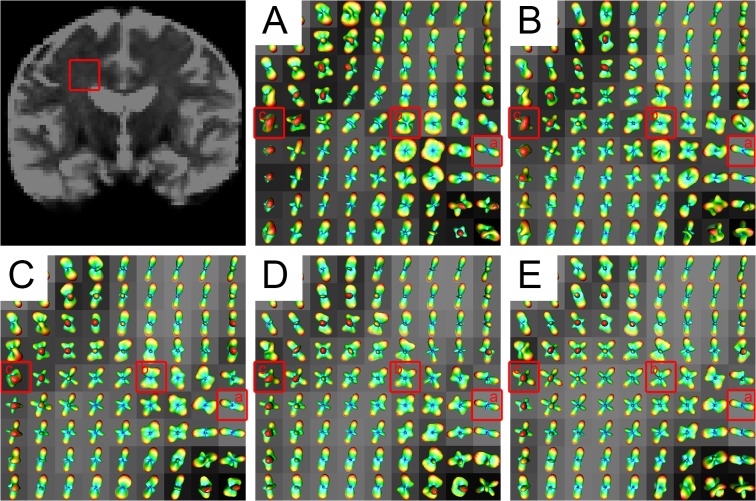
The diffusion ODFs of the DWI data from different b-values in the centrum semiovale. The b-values for (A)-(E) were 650, 1000, 1500, 2000, 2500 s/mm^2^, respectively. The top-left figure indicates the centrum semiovale by a red rectangle. The three specially marked voxels in (a)-(c) are given a closer look in Fig. 12.

**Fig 12:**
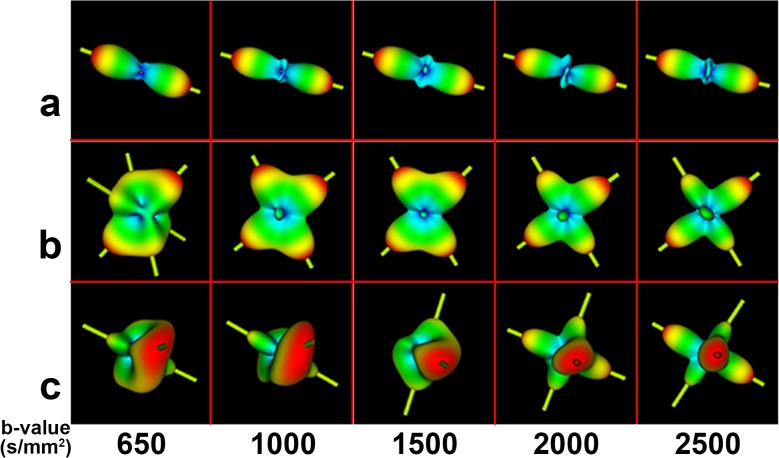
The diffusion ODFs of three voxels (those marked in Fig. 11A-C) at different b-values. The three voxels contain different numbers of fiber directions. Each row illustrates the ODFs of the same voxel at different b-values. The yellow sticks represent the principal peak(s) of the ODFs.

The centrum semiovale, which contains three types of crossing fibers [corona radiate (CR), superior longitudinal fasciculus (SLF), and the striations of the corpus callosum (CC)] [26], has been widely used as the area of focus in studies assessing modeling results with respect to b-values. Fig. 11 shows the ODFs of the centrum semiovale at five b-values for S1 and Fig. 12 shows a closer look at the ODFs of three voxels (also marked in Fig. 11) in the centrum semiovale at five b-values. Fig. 11 shows that the ODFs became sharper and that more fiber directions were detected with an increase in b-value. The crossing fibers that formed small angles could be discriminated more easily at higher b-values. Single-fiber situations, such as that in Voxel a of Fig. 12 was able to be reconstructed successfully by all b-values. Values of b = 2000 s/mm^2^ and above discriminated most of the two-way and three-way crossing-fiber situations. Figs. 11 and 12B show that most of the two-way intersecting fibers could be detected when the b-value was greater than 1500 s/mm^2^ and Fig. 12C shows that three-way crossing fibers, such as Voxel c, could be discriminated by b-values = 2000 s/mm^2^ and beyond.

### 3.6 Fiber ODFs in the centrum semiovale using CSD

**Fig 13:**
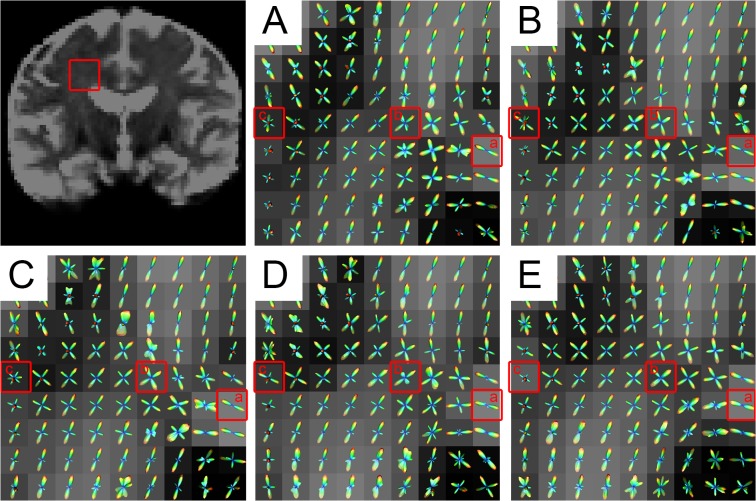
The fiber ODFs of the DWI data from different b-values in the centrum semiovale. The b-values of (A)-(E) = 650, 1000, 1500, 2000, 2500 s/mm^2^, respectively. The top-left figure indicates the centrum semiovale by a red rectangle. The three specially marked voxels in (a)-(c) are given a closer look in Fig. 14.

**Fig 14:**
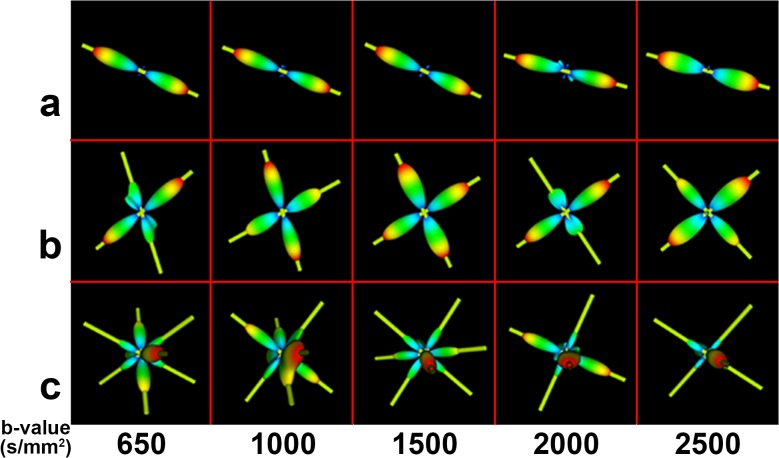
The fiber ODFs of three voxels (also marked in Fig. 13A-C) at different b-values. The three voxels contain different numbers of fiber directions. Each row illustrates the ODFs of the same voxel at different b-values. The yellow sticks represent the principal peak(s) of the ODFs.

We visually inspected the ODFs of the centrum semiovale obtained using CSD at five b-values. Fig. 13 shows the ODFs of the centrum semiovale at five b-values for S1 and Fig. 14 shows close-ups of the ODFs for three voxels in the centrum semiovale at five b-values. Despite the greater number of spurious peaks at higher b-values, the single-fiber situation was able to be reconstructed successfully by all five b-values (Fig.14A). Judging from Figs. 13 and 14B-C, most crossing-fiber situations were able to be resolved and the principal directions remained consistent when b = 2000 s/mm^2^ and 2500 s/mm^2^. Voxel b, which contains two-directional fibers, can be resolved when b = 1000 s/mm^2^ and above and the three-directional fibers in Voxel c can be clearly detected when b = 2000 s/mm^2^ and 2500 s/mm^2^.

## Discussion

By using in-vivo human brain DWI data collected utilizing a clinical MRI protocol that used five b-values (650, 1000, 1500, 2000, 2500 s/mm^2^), we systematically investigated the effects of b-value on the diffusion ODF and the fiber ODF, which represent two families of HARDI reconstruction methods, that is, the decomposition-based SPFI and the deconvolution-based CSD, respectively. A quantitative analysis and qualitative observations were combined to investigate the effects of b-value. First, the angular resolution of the diffusion ODF and the fiber ODF was quantified by calculating the FWHM index at different b-values. The results show that the FWHM decreased and the rate of change in the FWHM slowed down with an increase in b-value. The change in the FWHM between the b-values of 2000 s/mm^2^ and 2500 s/mm^2^ was less than 1°for both types of ODFs. The differences in FWHM for Subject One (S1) and for the group result were not significant between the b-values of 2000 s/mm^2^ and 2500 s/mm^2^ for either the diffusion ODF or the fiber ODF (Figs. 3–6). The angular differences in the ODF between each pair of b-values of 1500, 2000 and 2500 s/mm^2^ were similar and lower than the others in both single-direction and two-directional situations (Figs. 8 and 10). In the qualitative analysis, we inspected the ODFs in a fiber interweaving region to evaluate the capability of discriminating crossing fibers. Figs. 11 and 13 show that b = 2000 s/mm^2^ and above reveal most of the two-way or three-way crossing-fiber structures. Compared with b = 2000 s/mm^2^, b = 1500s/mm^2^ could not easily determine the multiple diffusion directions based on a usual way of computing local maxims of ODFs (Figs. 12 and 14). Inevitably, this inability to determine multiple diffusion directions would deteriorate the accuracy of fiber tracking and lead to false positive fibers or missing, but important, structural information. However we found an increase in spurious peaks in both the fiber ODFs and diffusion ODFs that were estimated with higher b-values because of increasing noise (Figs. 12A and 14A). Taking into consideration the SNR of the DWI data and the scanning time, b = 2000 s/mm^2^ appears to be an appropriate value for determining multi-way crossing fibers, judging by the results from the two classes of current HARDI reconstruction methods. These findings provide a useful reference for neuroscientists and clinicians to set appropriate scanning protocols for clinical HARDI applications.

The FWHM was used to represent the angular resolution of the ODFs quantitatively [26]. The more directed the fiber configuration in a voxel, the smaller the FWHM. The corpus callosum has been utilized as an alternative standard for the measurement of FWHM [26]. As shown in Figs. 3 and 5, both diffusion and fiber ODFs with higher b-values led to smaller FWHMs and narrower ODF glyphs. In the group results from the eight subjects (Figs. 4 and 6), the trend in which higher b-values yielded smaller FWHM was similar to that found for S1. The difference in the FWHM of the fiber ODF was only about 2 degrees between b = 650 s/mm^2^ and b = 2500 s/mm^2^ in the group results because the fiber ODF was sharper. However, the FWHM of the diffusion ODF was 67.7°at b = 650 s/mm^2^ compared with 56.9°at b = 2500 s/mm^2^ for the group results. These numbers imply that higher b-values can provide a better angular resolution and a sharper diffusion ODF. The better angular resolution can help to distinguish crossing fiber configurations with smaller crossing angles, and a sharper diffusion ODF contributes to achieving a more accurate tractography. The difference in the FWHM from b-values of 2000 s/mm^2^ to 2500 s/mm^2^ is smaller than 1°for both the diffusion and the fiber ODFs, an angular difference which can be regarded as insignificant compared with the error related to the MR noise and bias inherent to the HARDI finite sampling directions and ODF reconstruction. The differences in the FWHM between the b-values of 2000 and 2500 were not significant (Figs. 3–6). However, Figs. 3A and 5A also show that the FWHM-ODF curve for the single-direction situation began increasing when the angle subtended by the principal direction exceeded 60 degrees and 40 degrees, respectively. This may have resulted from differences in the information represented by the two different ODFs and from the decreased SNR. The diffusion ODF represents the motion of water molecules, which often, but not exclusively, diffuse parallel to the nerve fiber directions [22]. Therefore, diffusion ODFs can even represent the diffusion of water molecules perpendicular to the nerve fibers. Thus, the diffusion ODF value for the direction that is perpendicular to the principal peak may not be zero. In contrast, fiber ODFs describe the distribution of the nerve fiber directions within a voxel [22], and spurious peaks are a common problem for fiber ODFs [36]. Another possible explanation is that higher b-values induce more noise and an inadequate SNR can result in more and larger spurious peaks for both types of ODFs. From the above, it should be clear that there is a tradeoff between b-value and the SNR. Although better angular resolution can be achieved at higher b-values, the lower SNR can result in misestimation. Therefore, based on our results for the FWHM, b = 2000 s/mm^2^ seems to be an appropriate selection for ODF reconstruction in that it balances the SNR difficulties of high b-values with the weaknesses of low b-values.

The angular differences of the principal direction(s) extracted from the diffusion and fiber ODFs between different b-values were measured separately to investigate the appropriate b-value by identifying the one whose principal peak had a smaller change compared with the b-values around it. As shown in Figs. 8 and 10, for both the diffusion and the fiber ODFs, the angular differences between each pair of b-values of 1500 s/mm^2^, 2000 s/mm^2^ and 2500 s/mm^2^ were lower than the others in both single-direction and two-directional situations. Furthermore, the difference in the diffusion and fiber ODFs was lowest between b-values of 2000 s/mm^2^ and 2500 s/mm^2^. This indicates that ODFs reconstructed with b-values around 2000 s/mm^2^ begin to maintain consistent peaks. Higher b-values can facilitate the reconstruction of more precise and sharper ODFs, and sharper ODFs are helpful for accurately extracting the principal direction and decreasing the angular difference. Although lower angular differences can be achieved at higher b-values, their impact is swamped by the noise under realistic acquisition conditions. Therefore, b = 2000 s/mm^2^ again seems to provide a good compromise between the angular difference and the SNR.

Because no previous gold standard had been established, some previous experiments assessed the ODFs by visual inspection when using in-vivo human brain data [27, 28]. The centrum semiovale is a complex region which contains three-way crossing fibers [26] and hence has been routinely selected as the ROI for evaluating the performance of HARDI methods in many studies. In Prckovska et al. [28], the centrum semiovale was adopted to compare QBI and the diffusion orientation transform [37] at various b-values. Wedeen et al. [38] used it to evaluate DSI, and Kuo et al. [27] compared DSI and QBI by accessing the ODFs of the centrum semiovale. In our study, we separately investigated diffusion and fiber ODFs, which are estimated from two different categories of HARDI methods, in the centrum semiovale. Both diffusion and fiber ODFs reconstructed with b-values lower than 2000 s/mm^2^ revealed less anatomical information or misestimated fiber directions (Figs. 11 and 13). This underestimation should be avoided to allow the subsequent tractography to be accurate. Voxel a, which contained a single-direction fiber, was able to be solved with all five b-values for both the SPFI and the CSD methods. However, at increasing b-values, peaks that were perpendicular to the principal direction appeared in both the diffusion and the fiber ODFs. This may have been because the diffusion ODF represents the diffusion of water molecules in q-space and because the decreased SNR led to more spurious peaks. Although the ODF was sharper at higher b-values, Voxel b, which contained two direction fibers, was able to be solved when b ≥ 1000 s/mm^2^. The more complicated population in Voxel c, which contained 3 direction fibers, could not be revealed until the b-value reached 2000 s/mm^2^. However, Alexander and Barker [23] suggested that b = [2200, 2800] s/mm^2^ is optimal for two-fiber situations in their simulation study, and our results are consistent with this conclusion. We suggest that b = 2000 s/mm^2^ is the basic requirement for current HARDI methods on in-vivo human brain data collected by clinical MRI protocols on a 3T scanner.

Note that the selection of the appropriate b-value for HARDI reconstruction can be affected by a number of factors, including imaging parameters and post-processing parameters [26]. Increasing the number of diffusion gradients may improve the accuracy and angular resolution of the ODF at the same b-value. Using a high order of spherical harmonics in the reconstruction may enhance the angular resolution of the ODF when the sampling gradients are sufficient. Additionally, although the two examined reconstruction methods, the decomposition-based SPFI method and the deconvolution-based CSD method, are well-established methods, other algorithms may generate different results and thus slightly change the proposed b-value. Indeed, in the current situation in which newly emerging human brain projects are being launched to collect clinical MRI data on a large scale, we believe that our finding will be a useful reference for setting the protocols for HARDI imaging. With the development of improved HARDI techniques, new reconstruction methods will also emerge and the optimal b-value should be expected to evolve.

## Conclusions

The FWHM of the diffusion and fiber ODFs decreased with increasing b-values for both Subject One (S1) and the group results. The differences in the FWHM of the diffusion ODF and the fiber ODF between the b-values of 2000 s/mm^2^ and 2500 s/mm^2^ were not significant. The angular differences between different b-values around 2000 s/mm^2^ began to remain consistent and were lower than the angular differences for b-values below that level for both the diffusion and fiber ODFs. In conclusion, taking into consideration the SNR of the diffusion-weighted data and the acquisition time, b = 2000 s/mm^2^ is the appropriate choice for diffusion and fiber ODFs reconstruction using current HARDI methods on clinical data. Our study can provide a useful reference for researchers to set appropriate b-values for clinical HARDI data collection.
